# Predictive Models to Determine Imagery Strategies Employed by Children to Judge Hand Laterality

**DOI:** 10.1371/journal.pone.0126568

**Published:** 2015-05-12

**Authors:** Steffie Spruijt, Marijtje L. A. Jongsma, John van der Kamp, Bert Steenbergen

**Affiliations:** 1 Radboud University Nijmegen, Behavioural Science Institute, Nijmegen, The Netherlands; 2 VU University Amsterdam, Research Institute Move, Amsterdam, The Netherlands; 3 University of Hong Kong, Institute of Human Performance, Hong Kong, China; 4 Australian Catholic University, School of Psychology, Melbourne, Australia; University of Reading, UNITED KINGDOM

## Abstract

A commonly used paradigm to study motor imagery is the hand laterality judgment task. The present study aimed to determine which strategies young children employ to successfully perform this task. Children of 5 to 8 years old (N = 92) judged laterality of back and palm view hand pictures in different rotation angles. Response accuracy and response duration were registered. Response durations of the trials with a correct judgment were fitted to a-priori defined predictive sinusoid models, representing different strategies to successfully perform the hand laterality judgment task. The first model predicted systematic changes in response duration as a function of rotation angle of the displayed hand. The second model predicted that response durations are affected by biomechanical constraints of hand rotation. If observed data could be best described by the first model, this would argue for a mental imagery strategy that does not involve motor processes to solve the task. The second model reflects a motor imagery strategy to solve the task. In line with previous research, we showed an age-related increase in response accuracy and decrease in response duration in children. Observed data for both back and palm view showed that motor imagery strategies were used to perform hand laterality judgments, but that not all the children use these strategies (appropriately) at all times. A direct comparison of response duration patterns across age sheds new light on age-related differences in the strategies employed to solve the task. Importantly, the employment of the motor imagery strategy for successful task performance did *not* change with age.

## Introduction

A classic paradigm to study mental imagery of body parts is the hand laterality judgment (HLJ) task [1], in which participants make forced-choice judgments of whether pictures of hands display a right or a left hand. Participants can employ different mental imagery strategies to successfully solve the HLJ task. First, participants can imagine mentally rotating *their own hand* into the same position as the displayed hand, but without actually producing that movement. This strategy involves a first person or egocentric perspective, and is typically referred to as motor imagery [2–4]. Motor imagery is a cognitive process that involves the internal simulation of a movement without actually performing it [5–7]. The imagined hand rotation is presumed to exploit a motor representation for hand movements, and is therefore subject to the same constraints as actual hand movements [8]. Second, the HJL task can also be performed using strategies other than motor imagery. In particular, participants can mentally rotate the hand from a third person or allocentric perspective. Rather than exploiting a motor representation of hand movements, this strategy treats the hand like any other detached object. Put differently, within this strategy mental rotation is not constrained by or grounded in the motor system. This strategy is often referred to as visual imagery [2, 3, 8–10]. In the current study we are mainly interested in discriminating between mental imagery that is constrained by the motor system, and mental imagery that is not. We will therefore use the labels motor imagery and non-motor imagery.

Previous studies that examined the HLJ task in 5- to 12-year-old children have generally shown that HLJ task performance is affected by motor constraints, thus implying motor imagery is used to successfully solve the task [11, 12]. However, most of these studies were not specifically aimed at determining whether HLJ task performance can also be understood using alternative non-motor imagery strategies, and the age-related differences therein. The purpose of the present study is therefore to determine whether children of 5 to 8 years old indeed engage in motor imagery or whether they adopt non-motor imagery to perform the HLJ task. In doing so, we also aimed to address age-related differences in the imagery strategies that children employ. To accomplish these aims, we used an innovative method to discriminate between motor and non-motor imagery strategies. We developed a-priori sinusoid models that reflect the different strategies and examined how well they could predict actual HLJ task performance.

**Fig 1:**
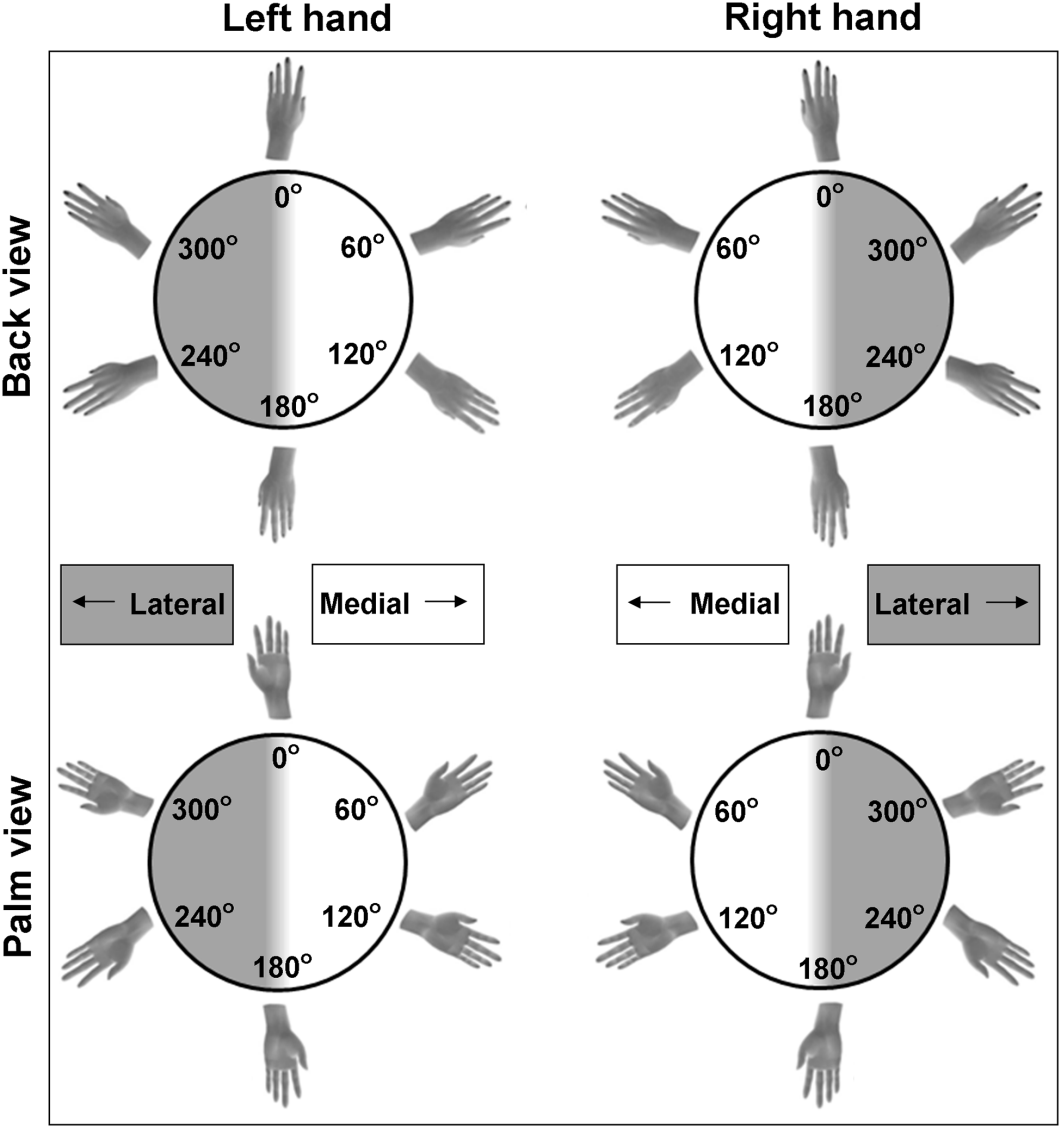
Stimulus Set. Hand stimuli (right and left hands; back and palm view) for the different rotation angles (0°; 60°; 120°; 180°; 240°; 300°). Rotation angles between 0° and 180° represent medial rotations; rotation angles between 180° and 360° represent lateral rotations, irrespective of hand.

In the HLJ task, left and right hand pictures [1] are displayed in different angles of rotation (i.e., showing hand rotations varying between 0° with finger pointing up to 180° with fingers pointing down), in different directions (i.e., showing medial rotations with the fingers pointing towards the midline of the body or lateral rotations with the fingers pointing away from the midline), showing either the palm or back of the hand. Fig 1 illustrates a standard set of stimuli presented in the HLJ task. Mental imagery performance is commonly evaluated using response accuracy and response duration as dependent variables [13, 14, 15].

The response durations for mentally rotating an object are systematically affected by rotation angle, with larger angles relative to the neutral 0°–rotation resulting in longer response durations, up to a maximum for the 180°–rotation [16–19]. Although the *rotation angle* can affect response duration irrespective of whether a non-motor imagery or a motor imagery strategy is used, the *direction of rotation* is presumed to only affect response durations for motor imagery strategies. In the case of a non-motor imagery strategy, hands that are rotated medially or laterally result in the same response durations as long as the rotation angle is the same [9]. In other words, a pattern of response durations that is symmetric around the 0°–rotation indicates a non-motor imagery strategy. By contrast, as motor imagery is subject to the same constraints as actual performance, the duration to mentally rotate one’s own hand to a biomechanically ‘awkward’ lateral posture (i.e., rotating the hand away from the central body axis) results in prolonged durations compared to rotating one’s hand towards a more ‘comfortable’ medial posture (i.e., rotating the hand towards the medial body axis) [16, 20–23]. Thus, a pattern of response durations that is asymmetric around the 0°–rotation indicates a motor imagery strategy [9]. Another indication for the involvement of motor imagery is the observation that the effects of rotation angle and direction of rotation on *actual* hand movement durations depend on the posture of the participants’ hands [16]. Parsons [16] showed that with the back of the hand facing upwards, physical hand rotation durations increase with rotation angle, with only small differences between lateral and medial rotations. In contrast, with the palm of the hand facing upwards, the differences between lateral and medial rotations are much more pronounced [16]. Accordingly, when rotation direction similarly affects the actual and imagined movement responses, the use of a motor imagery strategy is indicated. In sum, motor and non-motor imagery strategies in the HLJ task can be discriminated by considering the effects of rotation angle and direction of rotation on imagery response durations and by determining the differences in the response duration patterns for back and palm views.

Previous studies using the HLJ task in children have examined the effects of rotation angle and the direction of rotation on response durations. It was found that the laterality judgments for back and palm view hands by 5- to 12-year-old children are a function of rotation angle [10, 13, 24, 25] and the direction of the rotation [8, 11, 12, 14, 15, 26–28]. This suggests that primary school children, taken as a group, indeed use motor imagery to solve the HLJ task. Three studies directly considered age-related differences in children’s HLJ task performance. Krüger and Krist [15] reported that in contrast to 7-year-olds, the effect of motor constraints was “not so distinct” (p. 256) in 5-year-olds, as only the response durations for right hand pictures were affected by the direction of rotation. Similarly, Toussaint, Tahej, Thibaut, Possamai and Badets [29] recently indicated that the difference in response durations between lateral and medial rotations was larger in 8-year-olds than in 6-year-olds. Although these observations suggest that the contribution of motor imagery in judging hand laterality progresses between 5 and 8 years of age, they do not address the age at which children start to rely on motor imagery relative to non-motor imagery strategies. Butson, Hyde, Steenbergen and Williams [28] suggested that biomechanical constraints affect response durations in children of 8, 9 and 11 years old that were able to perform the task correctly, but not in children of 7 and 10 years old. Based on additional results that biomechanical constraints were reflected in response accuracy, they concluded that most 7- to 11- year olds were engaged in motor imagery to perform the HLJ task, but left for future research to designate younger children’s use of motor imagery strategies.

The present study uses a-priori defined predictive sinusoid models that predict the changes in response duration patterns as a function of either the rotation angle (H1) or as a function of the rotation angle and direction of rotation (H2). These two models thus predict response duration patterns that would arise from employing either a non-motor or a motor imagery strategy to solve the HLJ task. These models were validated in a pilot experiment with adults, which confirmed that they can indeed discriminate between the two imagery strategies (see S1 Appendix). Examining the fit between the model predictions and actual response duration patterns for 5- to 8-year-old children allows us to determine the imagery strategy that children use to perform the HLJ task, and consequently, the age-related differences therein.

### The sinusoid models

**Fig 2:**
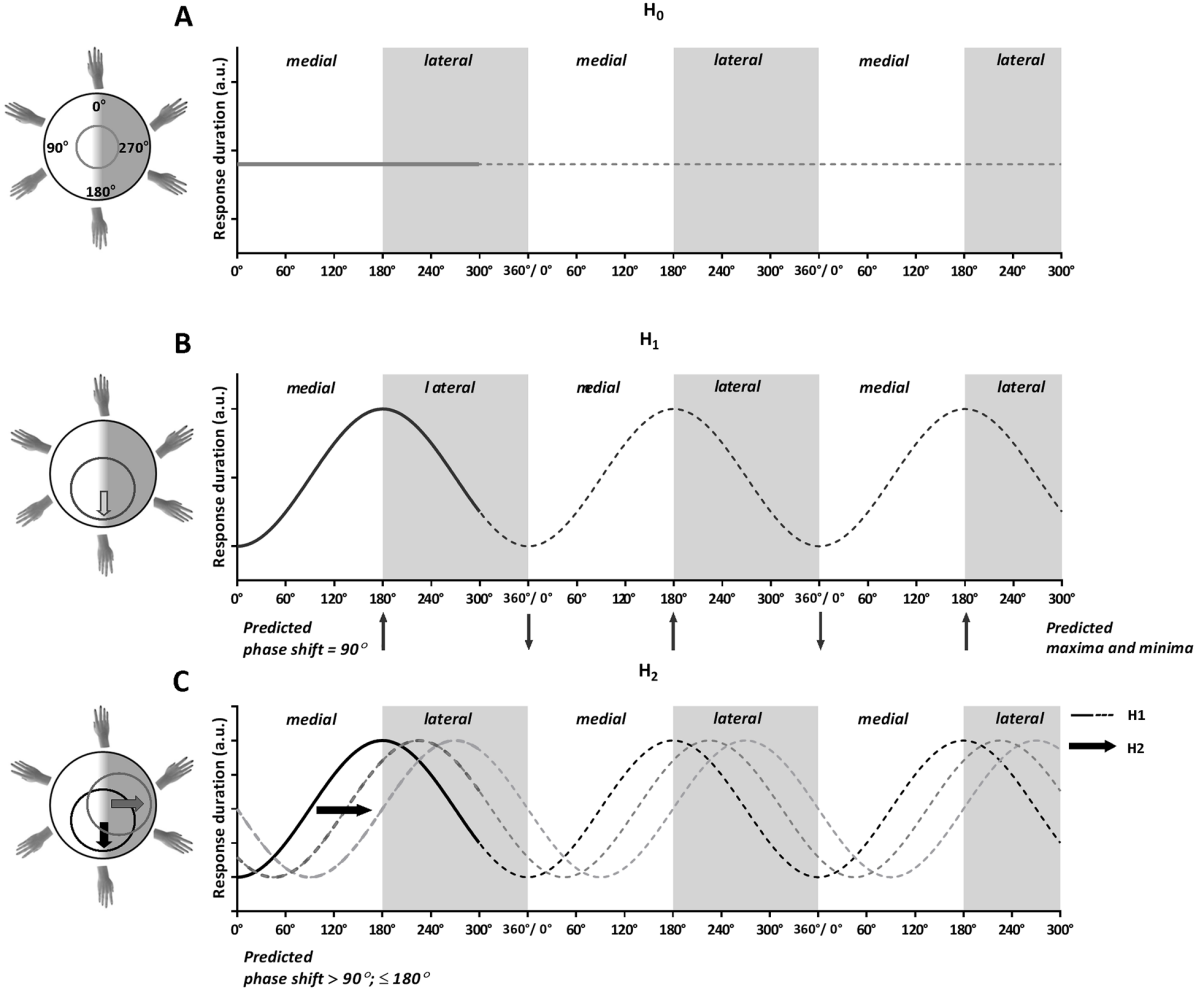
Modeled Response Duration Patterns. *Left*: Modeled distribution of response durations. The y-axis represents rotation angle, ranging from up (0°) to down (180°). The x-axis represents the direction of rotation, ranging from medial (white area) to lateral (grey area). The distance from the center of the axes to the line represents the response durations. *Right*: Modeled response duration curve over different angles of rotation. The solid lines represent the modeled curves (first wavelength). The dotted lines (second and third wavelength) were added to better visualize the modeled curves. *A)* Depicts the H0: no effect of rotation angle or direction. Amplitude = 0. *B)* Depicts the H1: with an increase in rotation angle, there is an increase in response duration. Phase shift = 90°. *C)* Depicts the H2: The H1 curve (black line) is shifted to the right, representing an increase in response duration as a function of a rotation in the lateral direction (grey lines). Phase shift >90°; ≤180°.

Because hand pictures were rotated in a flat surface in a 360° full circular fashion, sinusoid models were used to predict response duration patterns as a function of rotation angle. The first model predicts that neither changes in rotation angle, nor changes in direction of rotation systematically influence the response duration patterns. This H0 model is described by a sinusoid with amplitude 0 (response duration = amplitude * sin (angle—phase shift) + intercept). This is graphically represented by a straight, horizontal line (Fig 2A). As the response durations do not vary as a function of rotation angle or the direction of rotation, the H0 model represents a performance strategy other than mental imagery, such as the application of an abstract rule or identification on the basis of idiosyncratic visual cues (as suggested by ter Horst and colleagues [20]).

The second model predicts changes in the response duration pattern that are symmetric around the 0°–rotation. In this H1 model, response durations change *only* as a function of rotation angle. An increase in rotation angle from 0° up to 180° results in longer response durations, irrespective whether the rotation is medially or laterally (Fig 2B). This H1 model is described as a sinusoid with a phase shift of 90° (response duration = amplitude * sin (angle—phase shift) + intercept). The phase shift of 90° reflects that response durations are shortest (i.e. fastest responses) at a 0°–rotation and largest when the hand stimuli are rotated over 180° (Fig 2B). As the model only includes an effect of rotation angle, and not the direction of rotation, the model represents a non-motor imagery strategy. The amplitude reflects the strength of the effect of rotation angle, i.e. the higher the amplitude, the stronger the effect of rotation angle on response duration patterns.

The third model predicts the asymmetric effect on imagery response durations around the 0°-rotation related to the direction of rotation. The H2 model predicts that lateral hand rotations result in longer response durations compared to medial rotations. The H2 model comprises a similar sinusoid, but with an additional phase shift that is larger than 90° and smaller or equal to 180° (response duration = amplitude * sin (angle—phase shift) + intercept). The response durations decrease for medially rotated hands and increase for laterally rotated hands (Fig 2C). The phase shift can vary from 90° (i.e., response durations are predominantly affected by rotation angle, cf. H1 model) up to 180° (i.e., response durations are predominantly affected by the direction of rotation). As the H2 model encompasses the direction of rotation, it represents a motor imagery strategy: mental rotation of the hand is subject to the same motor constraints as actual hand rotations (i.e., more awkward rotations take more time) [16, 20]. Note that this model includes a scenario involving an effect of direction of rotation only (i.e., phase shift = 180°, thus without an additional effect of the rotation angle).

### Current study

The current study examines the imagery strategies that children between 5 and 8 years of age use to solve the HLJ task. Prior work on the HLJ task in children mainly focused on general effects of the rotation angle and/or direction of rotation on imagery response durations. Only a few studies compared the effects between age groups, or determined the effects at one specific age. In the current study, we determine the combined effect of rotation angle and direction of rotation for the total group of children and for each individual child. Critically, we assess the phase shift parameter as this distinguishes the H1 model for non-motor imagery from the H2 model for motor imagery. Consequently, we can identify the involvement of motor imagery strategy to solve the HLJ task. Furthermore, regression analyses on the fitted parameters of the individual children allow establishing age-related differences in employed imagery strategies. We hypothesize that children’s HLJ task performance will be affected by both the rotation angle and the direction of rotation, indicating that they adopt a motor imagery strategy (indicated by the H2 model with a phase shift larger than 90° and smaller or equal to 180°). In line with observations in adults [16] we expect to find the direction of rotation effect to be more pronounced for judgments of palm view than for back view judgments (indicated by a larger phase shift). Finally, we expect more pronounced rotation direction effects with increasing age (indicated by larger phase shifts), as it was previously found that motor involvement increases between 5 and 8 years of age [15, 29].

## Methods

### Participants

A total of 92 right-handed, typically developing children between 5.2 and 8.9 years (M = 6.91; SD = 1.0) were recruited from mainstream primary schools in the Netherlands. Nonverbal intelligence quotient (IQ) was estimated using two subtests of the Dutch version of the Wechsler Nonverbal Scale of Ability, first edition [30]. Up to 7 years of age, children performed the Matrices and Recognition subtests, while the 8-year-olds performed the Matrices and Spatial Recognition subtests. The reported reliability of these subtests is considered sufficient for estimating IQ (Matrices: *α* = 0.77; Recognition: *α* = 0.64; Spatial Recognition: *α* = 0.74) [31]. The average IQ was 103 (SD = 13.9) and 45.7% of the participants was male.

#### Ethics Statement

The study has been approved by the local ethics committee of the Faculty of Social Sciences at the Radboud University Nijmegen (ECG2012-2402-018). Parents provided written informed consent prior to the experiment.

### Material and procedure

A computerized HLJ task was used, in which children judged whether a picture displayed a left or a right hand. The children were comfortably seated at a table, facing a laptop. They placed the left hand on a button at the left hand side and the right hand on a button at the right hand side. The hands were covered with a black cloth in order to prevent the children from watching their hands. The procedure was as follows. First, a white fixation cross was presented in the middle of the black screen for a random duration between 1000 and 1500 milliseconds. Subsequently, a picture of a hand was presented in the middle of the screen. The children were instructed to indicate whether the displayed hand was a left hand or a right hand by pressing the corresponding button as fast as possible. After the response was given, the picture disappeared and the fixation cross was again shown until the next stimulus was presented. The children were instructed that they were not allowed to make any hand and/or head rotations during the judgment.

The stimuli were pictures of left and right hands, rotated in six different rotations: 0°; 60°; 120°; 180°; 240°; 300° (Fig 1). Stimuli with a rotation angle of 0° showed the hand with the fingers pointing upwards, stimuli with a rotation angle of 180° displayed the hand with the fingers pointing down. Rotation angles for left hand stimuli were defined in a clockwise manner, while rotation angles for right hand stimuli were defined in a counter-clockwise manner. Consequently, stimuli with rotation angles between 0° and 180° were medially rotated and stimuli with rotation angles between 180° and 360° degrees were laterally rotated. Finally, the stimuli were presented in two different views. In the first block, the stimuli showed the back of the hands (Fig 1, top panels), while in the second block they showed the palm of the hand (Fig 1, bottom panels). Block order was the same for all children. Each unique stimulus was presented three times, resulting in 36 randomly ordered trials for each of two views. Six additional practice trials of different rotation angles were performed prior to the start of each block.

### Data analysis

#### Response accuracy

We first established whether or not the children performed the HLJ task above chance level. Based on a binomial distribution (*p* = 0.50 for each trial), individual performance was significantly above chance level when more than 23 out of 36 stimuli were correctly identified. Individual chance scores were determined for each view (back and palm) separately. Subsequently, we used analysis of variances to compare age and IQ scores of the children that were able to successfully perform the HLJ task to the children that did not successfully perform the task.

#### Response duration

The response durations were only analyzed for the children who performed above chance. In addition, as we were primarily interested in the strategies used to successfully perform the task, trials with an incorrect judgment were removed (i.e., 12% of the trials). Finally, outlier trials, which were defined as response duration < 250 ms or response duration > mean response duration + 3*standard deviation, were also excluded from further analysis (i.e., 2% of the trials). Response durations were averaged across three repetitions of each of the 12 stimuli for the back and palm view separately, resulting in four datasets of response duration for the six rotation angles; a set for the back view of the left hand; for the back view of the right hand; for the palm view of the left hand; and a set for the palm view of the right hand (see Fig 1).

Goodness of fit F-tests were used to model the response duration distribution as a function of the rotation angle and the direction of rotation in GraphPad Prism 6. First, the children performing above chance were analyzed as one group. That is, group curves were fitted on the individual averaged response durations of the six rotation angles per data set (back and palm view, left and right stimuli). The procedure comprised of three steps: i) Fitted group parameters (intercept, amplitude and phase shift) were compared between the four different data sets. When the curves of different sets shared all parameters, then the data sets were pooled for further analyses. ii) Next, it was tested if a sinusoid curve with amplitude > 0 (H1, H2) described the observed data better than a sinusoid with amplitude = 0 (H0). iii) If H0 was rejected, it was tested whether the phase shift is different from 90° (H1) or whether the phase shift is different from a value between 90° and 180° (H2). A Bonferroni correction was used (back and palm view, three models) that resulted in an alpha level of *p* = 0.0083. This analysis procedure using sinusoid models to discriminate between motor and non-motor imagery strategies was validated in a pilot experiment with adults (see S1 Appendix).

Moreover, for each individual child sinusoid curves were determined on the individual averaged response durations of the six rotation angles. This resulted in an intercept, amplitude and phase shift parameter for each child. By means of linear regression analyses we tested whether the individually fitted parameters could be predicted by age. These tests were performed for the back and palm view data separately.

## Results

### Response accuracy

Table 1 presents the response accuracy, age and IQ of the children performing at chance level and below chance level on the back and palm view. For both back and palm view it was found that the children who did correctly perform the task were older than the children who did not (Table 1; Back: *F*(1,91) = 10.5, *p* = 0.002, *η* ^*2*^ = 0.10; Palm: *F*(1,91) = 15.3, *p* = 0.000, *η* ^*2*^ = 0.15). Furthermore, IQ was significantly higher in the children who did perform the HLJ task above chance for palm view compared to the children who did not manage to solve the task systematically (Table 1; F(1, 90) = 7.91, *p* = 0.006, *η* ^*2*^ = 0.081). These differences in IQ were not found for back view (*p* = 0.24).

**Table 1:** Response accuracy, age and IQ for the children that performed at chance level and above chance level.

	Back view		Palm view	
	At chance	Above chance	At chance	Above chance
**Percentage of total group**	8.7%	91.3%	25%	75%
**Number of errors (SD)**	16.4 (0.89)	4.25 (0.37)	18.0 (0.56)	4.20 (0.33)
**Age (SD)**	5.88 (0.15)	7.01 (0.11)	6.26 (0.21)	7.13 (0.11)
**IQ (SD)**	97.6 (4.3)	104 (1.5)	96.4 (2.4)	105 (1.7)

### Response duration

**Fig 3:**
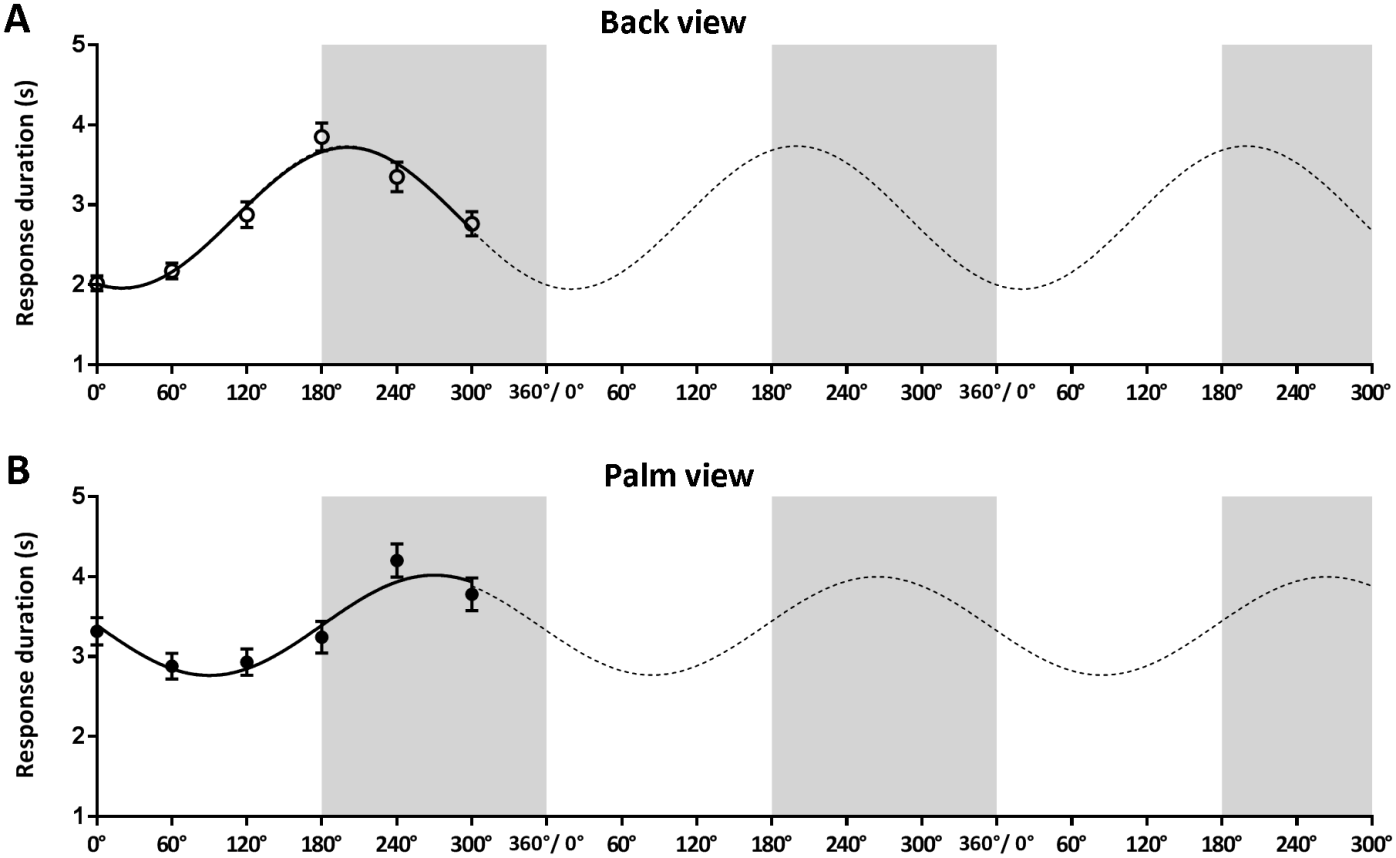
Response Durations Total Group. Response duration as a function of rotation angle. The solid lines represent the fitted sinusoid curves for the observed response durations (first wavelength). The dotted lines (second and third wavelengths) were added to better visualize the fitted curves. The data points in the first wavelength represent the mean response durations and standard error of means per rotation angle. Grey areas mark laterally rotated stimuli. *A)* Back view; *B)* Palm view.

For the group that performed above chance, the fitted group parameters did not significantly differ for the back view stimuli of the left and right hand; hence the two data sets were pooled. This resulted in the following group model: response duration (back view) = 0.8801 * sin(angle—110.4°) + 2.838 (Fig 3A). As shown in Table 2, the fitted phase shift parameter (110.4°) was significantly larger than 90° and significantly smaller than 180° (consistent with the H2 model). Hence, the resulting sinusoid shows that both the rotation angle and the direction of rotation affected the response durations when judging hands from the back.

**Table 2:** Fitted Parameters Total Group.

Fitted parameter	Tested against	Back view results			Palm view results		
		*F (*1,501*)*	*p*	*η* ^*2*^	*F (*1,411*)*	*p*	*η* ^*2*^
**Amplitude**	≈ 0	104	0.000*	0.38	49.0	0.000*	0.26
**Phase shift**	= 90°	12.6	0.000*	0.071	48.6	0.000*	0.26
**Phase shift**	= 180°	91.1	0.000*	0.35	0.51	0.48	0.004

F-Tests of goodness of fit for the fitted parameters for back and palm view.

* Significant (*p* < 0.0083; Bonferroni corrected)

Also, for the palm view stimuli, the fitted group parameters did not significantly differ between left and right hand stimuli. The two data sets were therefore pooled. This resulted in the following model equation for the total group: response duration (palm view) = 0.6392 * sin(angle—175.1°) + 3.390 (Fig 3B). The fitted phase shift parameter was significantly larger than 90°, but did not differ significantly from 180° (consistent with the H2 model). This indicates that the palm view judgments were only affected by direction of rotation, with minimum response durations at 90° (medial) and maximum response durations at 270° (lateral). The tests of fit parameters are listed in Table 2.

**Fig 4:**
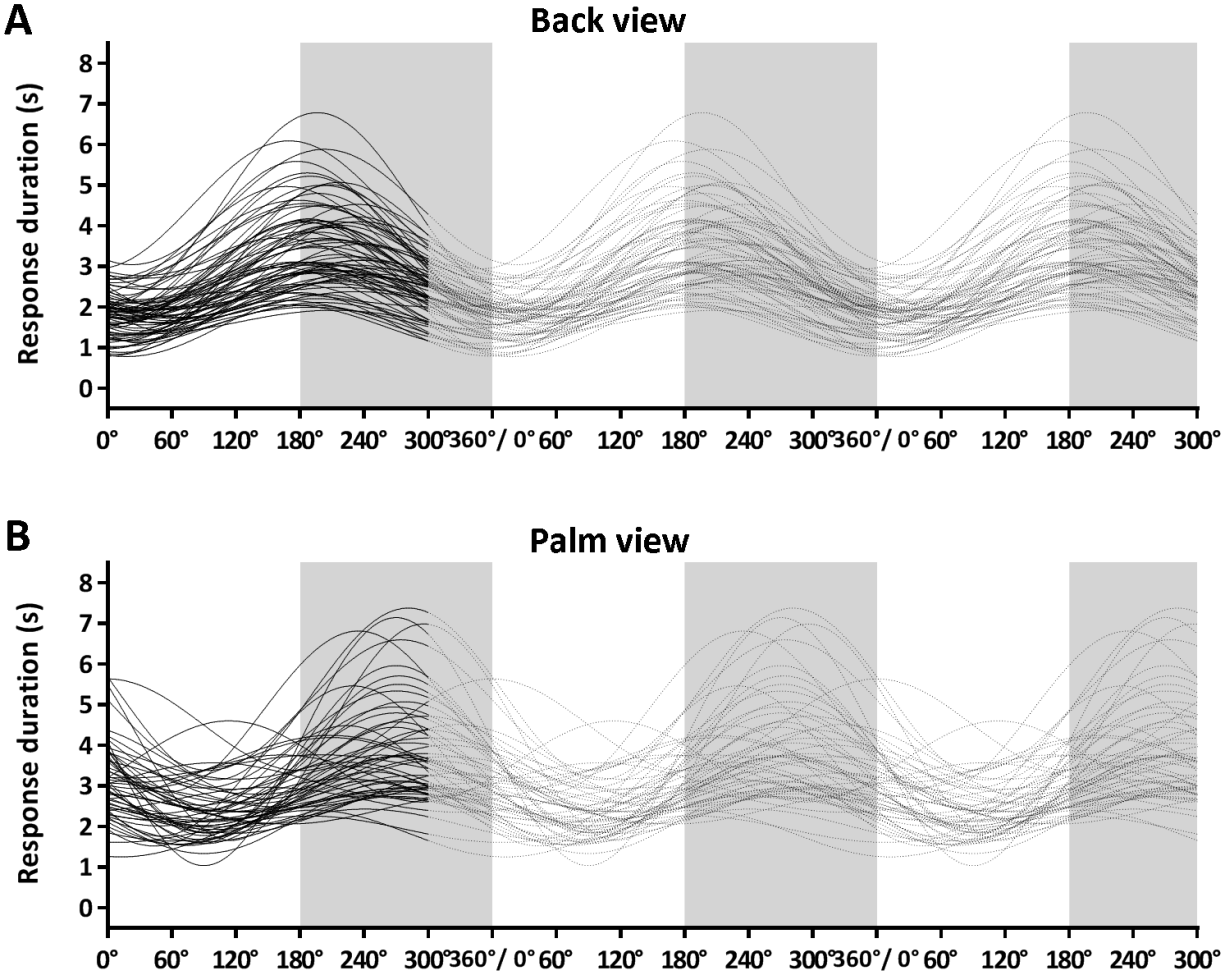
Response Durations Individual Children. Response duration as a function of rotation angle. The black lines represent the fitted sinusoid curves for all individuals (first wavelength). The grey lines (second and third wavelength) were added to better visualize the fitted curves. Grey areas mark laterally rotated stimuli. *A)* Back view; *B)* Palm view.

**Fig 5:**
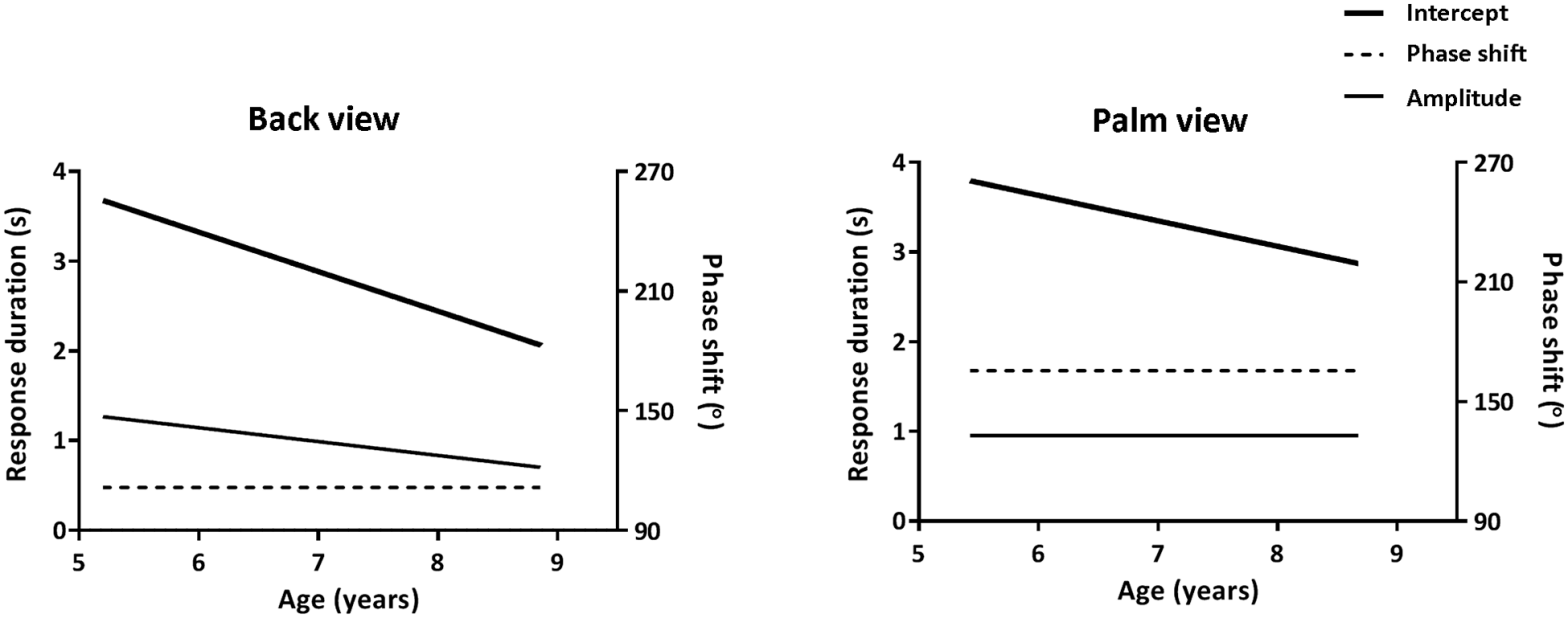
Fitted Parameters as a function of age. Linear regression on the fitted intercept (in seconds), amplitude (in seconds) and phase shift (in degrees) as a function of age (in years). *Left*: Back view; *Right*: Palm view.

The individually fitted curves for the back and palm view are presented in Fig 4. Table 3 presents the average fitted parameters for the individual participants. For the back view, the individual fit of four participants (5.2; 5.2; 6.9; 7.7 years old) did not reach significance. The individual fits did not result in a significant sinusoid curve for these individuals, reflecting that they did not use an imagery strategy. Therefore, they were excluded from the regression analysis on the fitted parameters. Five participants showing a trend towards significance (amplitude >0; *p* < 0.1) were included. Fig 5A shows that age both predicted the individually fitted intercept (*F*(1,79) = 11.3, *p* = 0.001, *R* ^*2*^ = 0.13) and amplitude (*F*(1,79) = 5.7, *p* = 0.019, *R* ^*2*^ = 0.07). However, the fitted phase shifts did not vary as a function of age (*p* = 0.30) (Fig 5A). For the palm view, the individual fit of seven participants (5.9; 5.9; 5.9; 7.1; 7.5; 7.6; 7.9 years old) did not reach significance and these individuals were excluded. Six participants showing a trend were included. It was found that age only predicted the fitted intercepts (*F*(1,61) = 4.3, *p* = 0.043, *R* ^*2*^ = 0.07), whereas the fitted amplitude (*p* = 0.20) and phase shift (*p* = 0.78) did not vary as a function of age (Fig 5B). Accordingly, Fig 5 illustrates that older children made faster judgments. The finding that the effects of rotation angle and direction of rotation (phase shift) were similar across age indicates that age did not seem to affect the strategies to solve the HLJ task.

**Table 3:** Average Fitted Parameters Individual Participants.

Back view results			Palm view results		
*Intercept (in s*.*) (SD)*	*Amplitude (in s*.*) (SD)*	*Phase shift (in degrees)(SD)*	*Intercept (in s*.*) (SD)*	*Amplitude (in s*.*) (SD)*	*Phase shift (in degrees) (SD)*
2.84 (1.19)	0.95 (0.58)	111 (24)	3.39 (1.03)	0.90 (0.62)	163 (54)

## Discussion

The present study examined the mental imagery strategies that children between 5 and 8 years of age use to successfully solve the HLJ task. The HLJ task is predominantly used to compare imagery ability between typically developing children and children with motor disorders [8, 10, 12, 14, 24–27]. Generally, these studies have interpreted HLJ task performance as a direct expression of the adoption of motor imagery. Similarly, differences in HLJ task performance between typically developing children of different age have been attributed to increases in the use of motor imagery [13, 15, 29]. However, motor imagery is not the only imagery strategy that can be adopted to perform the HLJ task [3, 10]. Hence, the present study attempts to account for the involvement of motor and non-motor imagery strategies in 5- to 8-year-old children who successfully solved the HLJ task. To this end, the response duration data were fitted to a-priori defined sinusoid models that describe response duration patterns for different imagery strategies, based on previous empirical findings. The models not only allow assessing the imagery strategy that is adopted by the children, but also potential age-related changes therein. In brief, the results demonstrated that for both back and palm view, children’s mental rotation was affected by biomechanical constraints (i.e., the H2 model was not falsified). This indicates that they used motor imagery in case they had performed the HLJ task successfully. Importantly, although the ability to correctly perform the task increased with age, there were no age-related differences in the motor involvement (i.e., the fitted phase shift parameter did not vary as a function of age). This underscores that once children successfully solve the HLJ task, the motor imagery strategy they employ remains unaltered until 8 years of age. We discuss these findings in more detail below, starting with the age-related differences in response accuracy.

The observed age-related increase in the capability to correctly perform the HLJ task is in line with previous work [13, 15, 29]. This indicates that from 5 to 8 years of age, older children become more proficient in correctly solving the HLJ task. Children that were able to do so on the palm view had higher IQ than children who were not. Hence, the development towards more proficient HLJ task performance may relate to a better understanding of task instructions, better working memory functioning, more abstract thinking and/or merely being able knowing left from right.

The improved capability in HLJ task performance with age, however, does not allow direct inferences regarding changes in the employed imagery strategy. Basically, the present findings indicate that children between 5 and 8 years old adopt motor imagery to successfully perform the task, irrespective of their age. First, the response duration patterns best fitted the H2 model, indicating that rotation angle and direction of rotation of the hands affected imagery performance durations in a similar way as durations of physical movement performance [16]. These effects of biomechanical constraints were found for both back and palm views, albeit that the direction of rotation had a stronger effect for palm view (as evidenced by the larger phase shift) [16]. In fact, the palm view showed an effect of direction of rotation only (i.e., the phase shift parameter did not differ from 180°). In other words, for rotations up to a maximum at 180° palm view judgments response durations did not increase as a function of increasing rotation angle. Nonetheless, the rotation direction predominantly affected response durations, with prolonged durations for laterally rotated stimuli (270°), compared to medially rotated stimuli (90°). This indicates that mental rotation predominantly involved motor imagery, without non-motor imagery contributions. However, we have to be careful in concluding that palm view judgments rely more on motor imagery than back view judgments, because in the current design the back and palm view blocks were not counterbalanced. Hence, any difference can also be attributed to order or learning effects.

The observed individual phase shifts in the back and palm view did not change as a function of age, indicating that there were no age-related differences in the employed imagery strategy. Moreover, the observed individual amplitude of the palm view did also not change with age. This indicates that the effects of rotation angle and direction of rotation on response duration patterns did not change with age. We therefore conclude that between 5 and 8 years of age, children adopted a similar strategy when successfully judging hand laterality. Yet, there was a clear age effect for response speed. The older children responded faster, as indicated by the intercept parameter that significantly decreased with age. This difference does not reflect changes in employed strategies, but can alternatively be explained by changes in information processing speed across age. Our findings diverge somewhat from previous studies that suggested that motor involvement increased with age between 5 and 8 years of age [15, 29]. Except for differences in stimulus sets (e.g., the inclusion of foot and/or non-body stimuli in previous work), a likely reason for this discrepancy is the methods used to pinpoint the adopted imagery strategy. Unlike previous approaches, in which effects of the rotation angle and direction of rotation of the hand stimuli were determined separately, the current approach takes the cumulative effects of these factors into account. This allows precise establishment of the imagery strategy adopted and the current evidence clearly indicates that the employed mental imagery is grounded in motor constraints, which remained the same across age. Importantly, a validation experiment in adults confirmed that the new approach can indeed distinguish between motor and non-motor imagery strategies (S1 Appendix). A final reason for the discrepancy between previous and current findings may be the inclusion of children that performed the HLJ task at chance level [29]. In fact, comparing the imagery strategies adopted by children who successfully identified the laterality of the hand stimuli with those of children who could not is an important issue for future work. Using the current a-priori defined modeling approach would allow assessing whether children that fail to correctly judge hand laterality employ different, less appropriate strategies (e.g., indicated by the phase shift parameter).

In sum, based on response *accuracy*, it is suggested that the ability to correctly perform the HLJ task increases with age. Notwithstanding these age-related differences, the response *duration* patterns indicated that when 5- to 8-year-olds successfully perform the HLJ task, they do this by using motor imagery. Although children do respond faster when they get older, notably, children’s motor imagery strategy does not change with age between 5 and 8 years.

## Supporting Information

S1 Appendix Validation of the sinusoid models approach. (DOCX)

S1 Fig Response Durations Validation Experiment. Response duration as a function of rotation angle. The solid lines represent the fitted sinusoid curves for the observed response durations (first wavelength). The dotted lines (second and third wavelengths) were added to better visualize the fitted curves. The data points in the first wavelength represent the mean response durations and standard error of means per rotation angle. Grey areas mark laterally rotated stimuli. *A)* Letters; *B)* Back view; *C)* Palm view.
(TIF)
