# Supplementary material for: Predictive Models to Determine Imagery Strategies Employed by Children to Judge Hand Laterality
Source: PLoS One. 2015 May 12;10(5):e0126568. doi: 10.1371/journal.pone.0126568 (PMC4428702; doi:10.1371/journal.pone.0126568)
Supplement: S1 Appendix — (DOCX) [file pone.0126568.s001.docx]

**S1 Appendix. Validation of the sinusoid models approach.**

To validate the a-priori defined predictive models, ten right-handed adults (mean age = 25.3 years; SD = 3.64; 3 male - 7 female) participated in an experiment that aimed to determine whether the a-priori models can indeed distinguish between non-motor and motor imagery strategies. The experimental procedure was similar to the procedure described in the manuscript (“Methods – Material and procedure”). In addition to the back and palm view stimuli, the adults also judged laterality of alphanumerical stimuli (the letter ‘F’; one block of 36 trials [as was also described in 14]). Block order was randomized. A group curve was fitted on the individual response duration patterns for the alphanumerical stimuli (letter) and for the hand stimuli (back and palm view) separately. These group fits were tested against the predictive models (H0, H1 and H2).

The alphanumerical data resulted in the following group model: Response duration (letter) = 0.219 * sin(angle – 101.6°) + 0.796. The fitted phase shift did not differ from 90^o^ (H1), indicating that response durations were increasing as a function of increasing rotation angle (up to 180^o^; S1 Figure A). In line with the expectation for mental rotation of non-body objects, this indicates that letters were judged using a non-motor imagery strategy. The following model was fitted on the back view data: Response duration (back view) = 0.270 * sin(angle – 90.4°) + 0.911. The fitted phase shift did not differ from 90^o^, indicating that direction of rotation did not affect response durations (S1 Figure B). Adults used a non-motor imagery strategy to judge laterality of back view hands. The palm view data resulted in the following group model: Response duration (palm view) = 0.190 * sin(angle – 149.3°) + 1.028. For the palm view hands the fitted group phase shift did not differ from 180^o^ (H2). S1 Figure C illustrates the effect of direction of rotation, as the response durations are shorter for medial rotations and prolonged for lateral rotations. It was thus shown that adults use motor imagery to judge laterality of palm view hands. In sum, we were able to distinguish between non-motor (H1) and motor imagery (H2) strategies by means of the sinusoid models. This confirms the validity of the current sinusoid models as a method to determine whether participants use non-motor or motor imagery strategies on mental rotation tasks.
