# Supplementary figures and images for: Predictive Models to Determine Imagery Strategies Employed by Children to Judge Hand Laterality
Source: PLoS One. 2015 May 12;10(5):e0126568. doi: 10.1371/journal.pone.0126568 (PMC4428702; doi:10.1371/journal.pone.0126568)

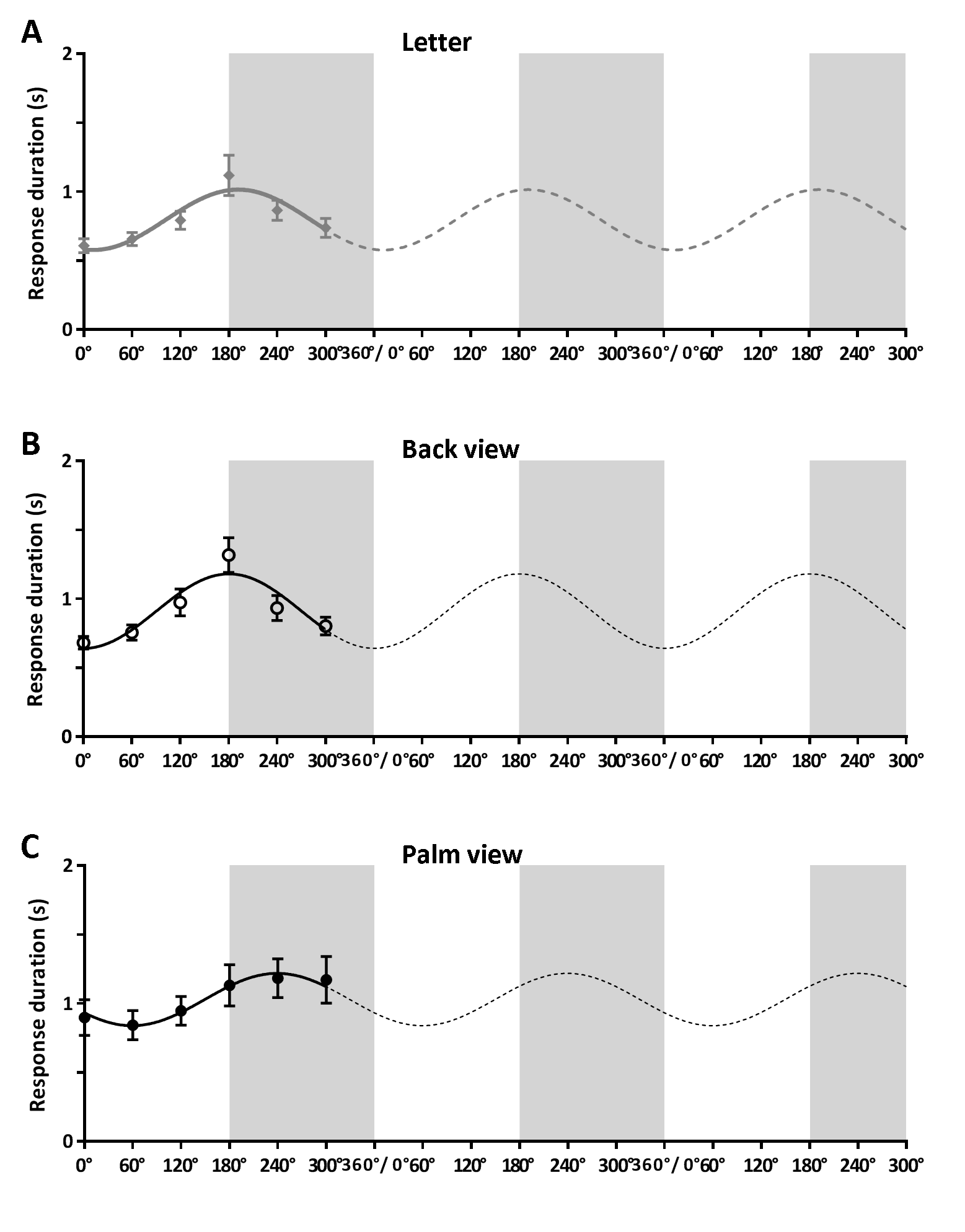

Supplement: S1 Fig — Response duration as a function of rotation angle. The solid lines represent the fitted sinusoid curves for the observed response durations (first wavelength). The dotted lines (second and third wavelengths) were added to better visualize the fitted curves. The data points in the first wavelength represent the mean response durations and standard error of means per rotation angle. Grey areas mark laterally rotated stimuli. A) Letters; B) Back view; C) Palm view. (TIF) [file pone.0126568.s002.tif]
